# The Neuroendocrine Functions of the Parathyroid Hormone 2 Receptor

**DOI:** 10.3389/fendo.2012.00121

**Published:** 2012-10-08

**Authors:** Arpád Dobolyi, Eugene Dimitrov, Miklós Palkovits, Ted B. Usdin

**Affiliations:** ^1^Neuromorphological and Neuroendocrine Research Laboratory, Department of Anatomy, Histology and Embryology, Hungarian Academy of Sciences, Semmelweis UniversityBudapest, Hungary; ^2^Section on Fundamental Neuroscience, National Institute of Mental Health, National Institute of HealthBethesda, MD, USA

**Keywords:** neuropeptide, thermoregulation, stress response, corticotropin-releasing hormone, somatostatin, neuroendocrine hypothalamic regulations, reproductive regulations, maternal adaptation

## Abstract

The G-protein coupled parathyroid hormone 2 receptor (PTH2R) is concentrated in endocrine and limbic regions in the forebrain. Its endogenous ligand, tuberoinfundibular peptide of 39 residues (TIP39), is synthesized in only two brain regions, within the posterior thalamus and the lateral pons. TIP39-expressing neurons have a widespread projection pattern, which matches the PTH2R distribution in the brain. Neuroendocrine centers including the preoptic area, the periventricular, paraventricular, and arcuate nuclei contain the highest density of PTH2R-positive networks. The administration of TIP39 and an antagonist of the PTH2R as well as the investigation of mice that lack functional TIP39 and PTH2R revealed the involvement of the PTH2R in a variety of neural and neuroendocrine functions. TIP39 acting via the PTH2R modulates several aspects of the stress response. It evokes corticosterone release by activating corticotropin-releasing hormone-containing neurons in the hypothalamic paraventricular nucleus. Block of TIP39 signaling elevates the anxiety state of animals and their fear response, and increases stress-induced analgesia. TIP39 has also been suggested to affect the release of additional pituitary hormones including arginine-vasopressin and growth hormone. A role of the TIP39-PTH2R system in thermoregulation was also identified. TIP39 may play a role in maintaining body temperature in a cold environment via descending excitatory pathways from the preoptic area. Anatomical and functional studies also implicated the TIP39-PTH2R system in nociceptive information processing. Finally, TIP39 induced in postpartum dams may play a role in the release of prolactin during lactation. Potential mechanisms leading to the activation of TIP39 neurons and how they influence the neuroendocrine system are also described. The unique TIP39-PTH2R neuromodulator system provides the possibility for developing drugs with a novel mechanism of action to control neuroendocrine disorders.

## Introduction

**Figure 1:**
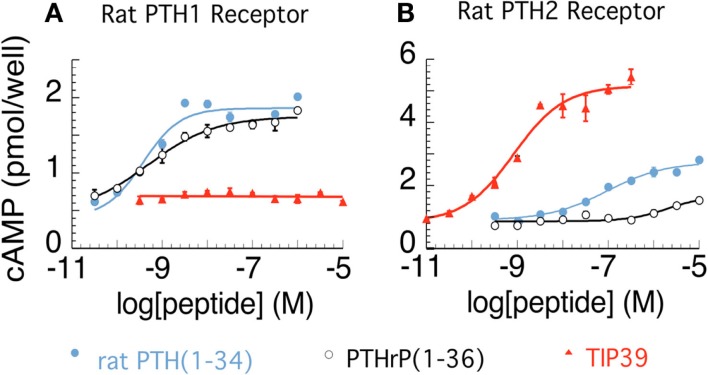
**Activation of rat parathyroid hormone 1 (PTH1) and PTH2 receptors**. cAMP accumulation is shown in relation to increasing concentrations of PTH, PTH-related peptide, and tuberoinfundibular peptide of 39 residues (TIP39) in COS7 cells expressing the rat PTH1R **(A)** and the rat PTH2R **(B)**, respectively. The figure was created from data presented previously (Hoare and Usdin, 1999; Hoare et al., 1999a; Usdin et al., 1999b).

The parathyroid hormone 2 receptor (PTH2R) is a member of the family B (type II) of G-protein coupled receptors. It was discovered based on its sequence similarity to other proteins belonging to this receptor family (Usdin et al., 1995). The novel receptor was named PTH2R because of its sequence similarity to the parathyroid hormone receptor and also because the human PTH2R can be activated by parathyroid hormone (Usdin et al., 2002). In rat, however, nanomolar concentrations of parathyroid hormone do not cause significant activation of the PTH2R (Hoare et al., 1999a; Figure 1). An additional difference between the parathyroid hormone 1 receptor (PTH1R) and the PTH2R is that the distinct polypeptide parathyroid hormone-related peptide is a co-ligand of the PTH1R but does not bind to the PTH2R (Hoare et al., 1999b). A breakthrough in the field of parathyroid hormone receptor research was the discovery of a novel peptide, tuberoinfundibular peptide of 39 residues (TIP39), an endogenous ligand of the PTH2R (Usdin et al., 1999b). TIP39 was purified from bovine hypothalamus based on its ability to elevate cAMP in a PTH2R-expressing cell line (Usdin et al., 1999b). TIP39’s sequence has very few amino acid residues in common with parathyroid hormone and parathyroid hormone-related peptide but it does have a similar three-dimensional structure to them (Piserchio et al., 2000). TIP39 is a high affinity and fully potent agonist for both the human and rodent PTH2R (Usdin et al., 1999b). Apart from elevating cAMP (presumably *via* Gs proteins), TIP39 was also shown to elevate intracellular Ca^2+^ levels (presumably *via* Gq proteins) in some cell types (Goold et al., 2001; Della Penna et al., 2003).

PTH2R expression is greater in the brain than in peripheral tissues based on Northern blot, *in situ* hybridization histochemistry and immunohistochemistry (Usdin et al., 1995, 1996, 1999a). In the periphery, its expression pattern was also very different from that of the PTH1R, as only a low level of expression was found distributed in the following places: pancreatic islet somatostatin synthesizing D cells, large vessels in bronchi, and the parenchyma in the lung, cardiac endothelium, a small number of cells associated with the vascular pole of renal glomeruli, spermatids in the head of the epididymis, atretic follicles of the ovary, chondrocytes in thyroid cartilage, a small number of cells in bone, and in some endocrine cells including thyroid parafollicular C cells, and some gastrointestinal peptide synthesizing cells. There is relatively little information on the effects of PTH2R signaling outside the brain and potential functions in the periphery are not discussed in this review.

Within the brain, the distributions of TIP39 axon terminals and PTH2R immunoreactivity show remarkable similarities as described below. Therefore, it was suggested that TIP39 is the endogenous ligand of the PTH2R. Consequently, it has been proposed that TIP39 and the PTH2R form a neuromodulator system in many brain regions. This is supported by the very similar phenotypes of mutant mice lacking either functional PTH2Rs or TIP39 or wild type (WT) animals administered a PTH2R antagonist as discussed below.

## The Distribution of the TIP39-PTH2R System

### Neurons expressing TIP39, the ligand of the PTH2R

**Figure 2:**
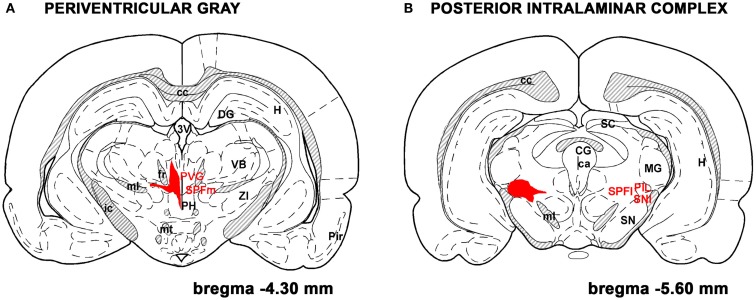
**TIP39 neurons in the subparafascicular area**. The subparafascicular area is a mediolaterally and to some degree rostrocaudally elongated region. Most TIP39 cells are located in its mediorostral portion, the periventricular gray of the thalamus **(A)** and in its caudolateral part, the posterior intralaminar complex of the thalamus **(B)**. The area where TIP39 neurons are distributed is shown in red. A few TIP39 neurons are scattered between these two regions below the fasciculus retroflexus and above the medial lemniscus. Additional abbreviations: ca, cerebral aqueduct; cc, corpus callosum; CG, central gray; DG, dentate gyrus; fr, fasciculus retroflexus; H, hippocampus; ic, internal capsule; MG, medial geniculate body; ml, medial lemniscus; mt, mamillothalamic tract; PH, posterior hypothalamus; Pir, piriform cortex; PVG, periventricular gray; SC, superior colliculus;SN, substantia nigra; SNl, substantia nigra, lateral subdivision; SPFl, lateral (parvocellular) subparafascicular nucleus; SPFm, magnocellular subparafascicular nucleus; VB, ventrobasal thalamus. The original drawings are modifications of panels from a rat brain atlas (Paxinos and Watson, 2007).

TIP39-expressing cells in the adult brain are restricted to the subparafascicular area of the thalamus and the medial paralemniscal nucleus of the pons as revealed by immunohistochemistry and *in situ* hybridization histochemistry (Dobolyi et al., 2003b) while the amygdala-hippocampal transitional zone also contains some TIP39 neurons during embryonic development (Brenner et al., 2008). The subparafascicular area TIP39 neurons were subdivided into the medially located group in the periventricular gray of the thalamus (PVG) and a laterally positioned group of the posterior intralaminar complex (PIL) of the thalamus (PIL; Dobolyi et al., 2010; Figure 2). Recent evidence supports the idea that the anatomical separation of these cell groups is concomitant with different functions. TIP39 appears in neurons in the PIL earlier during ontogenic development and largely disappears from them immediately after birth (Brenner et al., 2008). In turn, a marked induction of TIP39 in PIL cells but not in PVG cells can be observed in postpartum dams (Cservenak et al., 2010). Although the possibility that the PTH2R has another endogenous ligand apart from TIP39 is theoretically not excluded, the similarity in their distributions argues that TIP39 is available to activate PTH2Rs wherever they are present in the brain. Thus, neurons in the three sites of TIP39 expression would provide all information necessary to act *via* the PTH2R in the brain. Some evidence is available from lesion and tracer studies that TIP39 neurons in the subparafascicular area project to forebrain limbic and hypothalamic regions while TIP39 neurons in the medial paralemniscal nucleus provides TIP39 fibers to the hindbrain and spinal cord potentially affecting auditory and nociceptive functions (Dobolyi et al., 2003a; Wang et al., 2006b; Palkovits et al., 2010).

### TIP39 and the PTH2R in the pituitary and median eminence

Neither TIP39 nor PTH2R mRNA or protein were found in the pituitary (Usdin et al., 1999a; Dobolyi et al., 2002). In contrast, PTH2R-containing fibers were abundant in the external zone of the median eminence. PTH2R was found here in somatostatin fibers but not in fibers containing growth hormone (GH)-, gonadotropin-, or corticotropin-releasing hormones (CRH), or arginine-vasopressin (AVP; Dobolyi et al., 2006a). As opposed to the PTH2R, only a few TIP39-ir fibers were present in the median eminence. This is in fact the most striking difference between the otherwise very similar distributions of TIP39 axon terminals and the PTH2R.

### The similarities between the distribution of the PTH2R and TIP39 in the brain

The distribution of PTH2R fibers and PTH2R-expressing cells is generally similar in the brain and particularly so in the neuroendocrine hypothalamus (Wang et al., 2000; Faber et al., 2007). However, it has to be pointed out that while the labeling pattern of cell bodies provided by immunocytochemistry and *in situ* hybridization histochemistry was very similar in the rat (Wang et al., 2000), PTH2R-labeled cell bodies were mostly not visible by immunohistochemistry in the mouse, non-human primate and human (Faber et al., 2007; Bago et al., 2009). Nevertheless, *in situ* hybridization histochemistry and X-Gal histochemistry in mice expressing beta-galactosidase driven by the promoter of the PTH2R revealed a similar expression pattern of the PTH2R in these species as well (Faber et al., 2007; Bago et al., 2009). The finding that PTH2R-immunopositive fibers are often localized in the vicinity of PTH2R-expressing neurons suggests that these fibers may represent either axons or dendrites of local PTH2R-expressing neurons. Colocalization of the PTH2R with vesicular glutamate transporters suggests its axonal localization as described below in Section “Development of Fear.” Interestingly, the distributions, and even the subregional distributions of TIP39- and PTH2R-containing axon terminals are remarkably similar (Dobolyi et al., 2006a; Faber et al., 2007). Thus, TIP39 axon terminals and the PTH2R are co-distributed in the very same brain structures allowing the presynaptic modulation of PTH2R axon terminals. Therefore, an axo-axonal action of TIP39 is plausible (Dobolyi et al., 2010). However, there is no evidence available at present based on which the dendritic localization of the PTH2R can be excluded. In particular, electron microscopic investigation of the PTH2R has not been reported yet.

### The TIP39-PTH2R neuromodulator system in hypothalamic areas expressing neuroendocrine hormones

A high density of PTH2R-expressing cells and TIP39- and PTH2R-containing fibers is present in the medial preoptic nucleus and some surrounding parts of the medial preoptic area (Dobolyi et al., 2006a; Faber et al., 2007). A TIP39-PTH2R neuromodulator system is also abundant in the paraventricular and periventricular nuclei while other parts of the anterior hypothalamic region contain a lower density of labeling. Thus, TIP39 terminals are ideally positioned to influence somatostatin and CRH neurons. It has indeed been demonstrated that somatostatin neurons express the PTH2R (Wang et al., 2000; Dobolyi et al., 2006a) and that both TIP39- and PTH2R-containing terminals approximate CRH-expressing neurons in the parvicellular subdivision of the hypothalamic paraventricular nucleus (PVN; Bago et al., 2009; Dimitrov and Usdin, 2010). Similarly, in the tuberal region of the hypothalamus, the arcuate nucleus contains the highest density of PTH2R-expressing cells and TIP39- and PTH2R-containing fibers, providing an anatomical basis for influencing GH and prolactin release *via* GH-releasing hormone (GHRH) and dopamine neurons in the arcuate nucleus (Table 1)

**Table 1:** **The distribution of TIP39 axon terminals and the PTH2R in the nervous system**.

	Areas containing a high density of TIP39 fibers and PTH2R
Neuroendocrine hypothalamic areas	Medial preoptic area, paraventricular nucleus, periventricular nucleus, arcuate nucleus
Brain regions that potentially exert influence on the neuroendocrine system	Infralimbic cortex, lateral septal nucleus, the bed nucleus of the stria terminalis, medial and central amygdaloid nuclei, the vascular organ of the lamina terminalis, dorsomedial and perifornical hypothalamic nuclei, far-lateral and posterior hypothalamic areas, the medial subdivision of the supramamillary nucleus, premamillary nuclei, paraventricular, reuniens, posterior intralaminar, and subparafascicular thalamic nuclei, periaqueductal gray, lateral parabrachial nuclei, the locus coeruleus and subcoeruleus areas, and the nucleus of the solitary tract
Regions of the nervous system not directly involved in neuroendocrine regulations	Fundus striati, the medial subdivision of the medial geniculate body, tegmental areas of the midbrain and pons, deep layers of the superior colliculus, the external cortex of the inferior colliculus, the periolivary area, the nucleus of the trapezoid body, the superficial layers of the dorsal horn, the lateral cervical nucleus of the spinal cord, and some primary sensory neurons.

### TIP39 and the PTH2R in hypothalamic, limbic, and sensory brain regions that potentially exert influence on the neuroendocrine system

In the nervous system, TIP39 fibers and the PTH2R have a widespread distribution pattern (Wang et al., 2000; Dobolyi et al., 2003b; Faber et al., 2007; Bago et al., 2009). A number of brain regions known to affect the neuroendocrine system *via* neuronal projections to hypophysiotropic neurons were shown to contain a high density of PTH2R-expressing cell bodies as well as TIP39- and PTH2R-containing fibers. These brain regions include the medial prefrontal, especially the infralimbic cortex, the lateral septal nucleus, the bed nucleus of the stria terminalis, the amygdala, especially its medial and central nuclei, some midline and intralaminar thalamic nuclei, several hypothalamic nuclei, the periaqueductal gray, the lateral parabrachial nuclei, the locus coeruleus and subcoeruleus areas, and the nucleus of the solitary tract. Within the hypothalamus, the following nuclei contained a high level of TIP39 and PTH2R apart from the above mentioned neuroendocrine regions: the MnPO, the vascular organ of the lamina terminalis, the dorsomedial and perifornical hypothalamic nuclei, and some parts of the lateral hypothalamic area including the so-called far-lateral hypothalamus immediately next to the internal capsule, the medial subdivision of the supramamillary nucleus, the ventral, and dorsal premamillary nuclei, and the posterior hypothalamic nucleus, (Wang et al., 2000; Dobolyi et al., 2003b; Faber et al., 2007; Bago et al., 2009). In contrast, TIP39 and the PTH2R are scarce in a number of hypothalamic nuclei including the lateral preoptic area, the supraoptic, suprachiasmatic, and lateroanterior hypothalamic nuclei, the medial magnocellular part of the paraventricular nucleus, the ventromedial nucleus, and the medial and lateral nuclei of the mamillary body (Table 1).

### TIP39 and the PTH2R in regions of the nervous system not involved in neuroendocrine regulations

Elements of the TIP39-PTH2R neuromodulator system are also expressed in some brain regions that are not known to be directly or indirectly involved in neuroendocrine regulation. Thus, TIP39 as well as PTH2R fibers were abundant in the fundus striati, the medial geniculate body, tegmental areas of the midbrain and pons, the deep layers of the superior colliculus, the external cortex of the inferior colliculus, the periolivary area, the nucleus of the trapezoid body, the superficial layers of the dorsal horn and the lateral cervical nucleus of the spinal cord, and some cells in the dorsal root ganglia (Wang et al., 2000; Dobolyi et al., 2003b; Faber et al., 2007; Bago et al., 2009; Matsumoto et al., 2010). Several of these brain regions may participate in sensory, especially auditory information processing, which could represent non-neuroendocrine-related functions of the TIP39-PTH2R neuromodulator system. However, a potential role of TIP39 in the auditory stress pathway will be discussed later.

## Neuroendocrine Functions that May Involve the TIP39-PTH2R System

Based on the distribution of the TIP39-PTH2R system in the brain, its involvement in endocrine, limbic, nociceptive, and auditory functions have been hypothesized (Dobolyi et al., 2003a). These functions can of course be interrelated with each other. Initial studies in which PTH2Rs were activated with exogenous TIP39 as well as *in vitro* approaches provided some insights into the possible functions of the TIP39-PTH2R system. More recently, a selective and potent peptide antagonist of the PTH2R (Kuo and Usdin, 2007) and transgenic mice lacking functional TIP39 and PTH2R genes were developed (Fegley et al., 2008), which accelerated functional studies. It has indeed been established that the peptide neuromodulator system is involved in a variety of neuroendocrine functions including the stress response, thermoregulation, and prolactin release. Some evidence is also available for a role of the TIP39-PTH2R system in the regulation of AVP and GH release.

### Stress response

The stress response is a complex reaction of the organism to stimuli that threaten homeostasis. The stress response includes an altered psychological state called anxiety and physiological effects that include catecholamine and glucocorticoid release as final common pathways (Kvetnansky et al., 2009). The anxiety level and the hormone secretions interact to provide the organisms stress response.

#### The regulation of CRH function

The first functional evidence that the PTH2R might be involved in the regulation of CRH release came from an *in vitro* study. TIP39 increased CRH secretion from medial basal hypothalamic explants (Ward et al., 2001). In addition, intracerebroventricular injection of TIP39 dose-dependently increased the plasma adrenocorticotropin (ACTH) level at 10 min after injection in rat (Ward et al., 2001). In another study, local injection of TIP39 above the paraventricular hypothalamic nucleus (PVN) in mice also elevated plasma corticosterone levels in addition to increasing the number of pCREB-containing activated cells in and around the PVN (Dimitrov and Usdin, 2010). Most importantly, these effects of local TIP39 were not present in PTH2R knockout (KO) animals (Dimitrov and Usdin, 2010) excluding non-specific actions of TIP39 injection. These results suggest that the TIP39-PTH2R system is positioned and available to potentially modulate activation of the hypothalamus-pituitary-adrenal (HPA) axis. In fact, the activation of TIP39 neurons has been reported in some stress situations as described below in Section “The Activation of TIP39 Neurons.” Furthermore, the daily peak of the basal plasma corticosterone level was reduced in TIP39 KO mice suggesting that endogenous TIP39 plays a role in the circadian regulation of corticosterone levels (Dimitrov and Usdin, 2010). These findings are consistent with a high density of TIP39 fibers, PTH2R-expressing cells, and PTH2R-containing fiber terminals in the parvicellular subdivisions of the PVN (Faber et al., 2007). In fact, close apposition between CRH neurons and PTH2R-containing fibers has been demonstrated in mice (Dimitrov and Usdin, 2010) as well as in human (Bago et al., 2009).

#### The regulation of noradrenergic function

The involvement of the PTH2R in the regulation of the catecholamine systems is less well established even though TIP39 fibers are abundant in the locus coeruleus and subcoeruleus areas (Dobolyi et al., 2003b) where rostrally projecting noradrenergic neurons reside. Recent evidence brings up the possibility that TIP39 may interact with some noradrenergic pathways. TIP39 KO mice and WT mice injected with a PTH2R antagonist demonstrated selective impairment of memory performance during novelty-induced arousal (Coutellier et al., 2011a). Noradrenergic signaling has a biphasic, inverted U shaped, effect on cognitive functions (Arnsten, 2009). The impaired performance of mice without TIP39/PTH2R signaling was restored by propranolol, an antagonist of beta adrenoceptors, suggesting that PTH2R signaling influences the effect of novelty stress *via* an interaction with noradrenergic mechanisms (Coutellier et al., 2011a).

#### Stress-induced analgesia

In an experiment addressing the role of PTH2R signaling in stress-induced analgesia (SIA), hotplate tests were performed before and after an inescapable foot shock, used as the stressful stimulus (Dimitrov et al., 2010). As discussed in a later section, TIP39 KO and PTH2R KO mice as well as WT mice injected with a PTH2R antagonist had somewhat elevated pre-shock response latencies in the hotplate test. Following the foot shock, the response latencies increased in the WT mice as expected based on the phenomenon of SIA. Unexpectedly, a larger and dramatic increase was found in the response latency in mice without PTH2R signaling (Dimitrov et al., 2010). These findings suggest that signaling through the TIP39-PTH2R system may normally limit SIA.

Stress-induced analgesia induced by high intensity stressful stimuli, including inescapable foot shock, has been shown to have a predominant non-opioid component (Lewis et al., 1980; Terman et al., 1984). Indeed the opioid antagonist naloxone did not, but the CB1 cannabinoid receptor antagonist rimonabant did, decrease the SIA in WT mice (Dimitrov et al., 2010). The inhibitory effect of rimonabant on the SIA was much greater in the KO mice suggesting that the KO mice have greater endocannabinoid release or sensitivity and that endogenous TIP39, *via* the PTH2R, may negatively modulate release or an effect of endogenous cannabinoids during SIA.

### Release of arginine-vasopressin

The role of the PTH2R on the release of AVP was investigated by intracerebroventricular injection of TIP39 in rats (Sugimura et al., 2003). Reduced AVP levels were found in plasma 5 min following TIP39 administration. TIP39 also suppressed the plasma AVP increase following dehydration by water deprivation for 48h, hyperosmolality following i.p. injection of hypertonic saline, and hypovolemia following i.p. injection of polyethylene glycol. These inhibitory effects cannot be attributed to a decrease in the level of osmotic or hypovolemic stimulation because plasma Na^+^ and plasma total protein were not affected by the injection of TIP39 (Sugimura et al., 2003). The effect of TIP39 was also not a consequence of a change in blood pressure because injection of TIP39 produced a fall in mean arterial blood pressure, which would rather stimulate AVP secretion. In turn, the opioid receptor antagonist naloxone significantly reversed the inhibitory effect of TIP39 on dehydration-induced AVP release while it had no significant effect on the plasma AVP level when injected alone (Sugimura et al., 2003). These results suggest that TIP39 inhibits AVP release by central action – possibly *via* an opioid system but without hemodynamic or osmotic influence. The effect was rapid and did not last long suggesting that TIP39 may play a role in the dynamic regulation of AVP release. TIP39 and PTH2R are scarce in the supraoptic nucleus and the part of the PVN where magnocellular AVP neurons are located. Thus, TIP39 may exert its AVP release-inhibitory effect by acting through other hypothalamic nuclei. The hypothalamic arcuate nucleus is a candidate as it contains a high density of TIP39 and PTH2R immunoreactivity (Faber et al., 2007) as well as many opioid neurons, which are involved in the regulation of AVP release (Haaf et al., 1987; Heijning et al., 1991). Indirect action of TIP39 *via* hypothalamic angiotensin and atrial natriuretic peptide neurons affecting AVP release (Antunes-Rodrigues et al., 2004) is also conceivable.

### Growth hormone secretion

In a preliminary experiment, TIP39 injection into the lateral ventricle of male rats blocked the appearance of GH almost completely in plasma for the next 3 h (Usdin et al., 2003). This finding is consistent with anatomical data showing a high density of TIP39-containing fibers around somatostatin neurons in the periventricular hypothalamic nucleus. Somatostatin neurons in this area project to the median eminence and inhibit the release of GH (Luque et al., 2008). PTH2R expression was demonstrated on many of these somatostatin neurons in the rat (Usdin et al., 1999b) as well as in human (Bago et al., 2009) providing the anatomical basis for TIP39 stimulating the release of somatostatin, which in turn inhibits GH secretion (Luque et al., 2008).

## Physiological Actions of TIP39 not Directly Related to Its Neuroendocrine Effects

### Effects on the anxiety level

The anxiety levels of animals may also be affected by the TIP39-PTH2R system. In the elevated plus maze, a test of anxiety, intracerebroventricular injections of TIP39 in rats resulted in increased open arm entries and duration as compared to controls (animals receiving the inactive TIP (7–39) or saline) while no differences were observed between groups in the number of closed arm entries or total arm entries, suggesting an anxiolytic-like effect of TIP39 (LaBuda et al., 2004). The TIP39-PTH2R system in the infralimbic cortex, lateral hypothalamus, preoptic area, lateral septum, and the paraventricular thalamic nucleus could be involved in this action because TIP39 administration induced Fos activation in these brain regions (LaBuda et al., 2004). In the forced-swim test, a similar TIP39 administration reduced the duration of immobility, and increased the amount of climbing behavior (LaBuda et al., 2004) suggesting an anti-depressant-like action of TIP39. The phenotype of TIP39 KO mice is also consistent with an anxiolytic role of endogenous TIP39. TIP39 KO mice demonstrated increased anxiety in the shock-probe defensive burying test as compared to WT controls. In “standard/low stress” testing conditions, TIP39 KO mice did not differ from WT controls in the arm entries in the elevated plus maze or in the dark-light emergence test of spontaneous anxiety-like behaviors (Fegley et al., 2008). However, an increase in anxiety-like behavior became apparent in TIP39 KO mice that were tested in the elevated plus maze under conditions of mildly increased stress evoked by either brief prior restraint or bright illumination. These results are consistent with a role of endogenous TIP39 in limiting the consequences of stressful perturbations. Furthermore, mice lacking TIP39 or the PTH2R demonstrated increased anxiety- and depression-like behaviors 16–17 days but not 7–9 days after a foot shock in elevated-zero maze, open field, light-dark box and forced-swim tests (Coutellier and Usdin, 2011).

### Development of fear

Closely related to anxiety, fear has also been investigated in relation with the TIP39-PTH2R system using Pavlovian fear conditioning (Fegley et al., 2008). TIP39 KO mice showed more freezing than WT controls after only one tone-shock pairing during conditioning and, subsequently, more freezing during both tone- and context-recall tests (Fegley et al., 2008). However, based on a similar rate of decline in freezing responses to repeated tone presentation and a similar level of freezing during subsequent tone presentation, there did not appear to be an effect of TIP39 deletion on fear extinction learning or extinction recall, respectively (Fegley et al., 2008). Furthermore, foot shock conditioned fear recall was enhanced 14 days but not 6 days after the aversive stimulus in both TIP39 KO and PTH2R KO mice as compared to WT controls (Coutellier and Usdin, 2011). These results suggest that normal TIP39 signaling lessens the long-term consequences of a traumatic event while the absence of signaling *via* the PTH2R delays recovery. Since the amygdala is known to be involved in the fear response (LeDoux, 2003), the abundant TIP39-PTH2R system in the amygdala, especially in its central and medial nuclei (Faber et al., 2007), might be involved in these effects.

### Thermoregulation

Body temperature regulation is a fundamental homeostatic function that in homeothermic animals. Thermal information on environmental temperature sensed by skin thermoreceptors ascends to the MnPO in the hypothalamic preoptic area. GABAergic inhibitory neurons in the MnPO integrate this input with local thermal influences and project to the dorsomedial hypothalamic nucleus and the rostral medullary raphe region to restore homeostasis (Nakamura, 2011). Injection of TIP39 into the lateral ventricle increased the core temperature of WT mice while TIP39 injection had no effect in PTH2R KO mice, excluding the possibility of non-specific inflammatory actions (Dimitrov et al., 2011). Furthermore, PTH2R KO mice had impaired heat production upon cold exposure, but no change in basal temperature and no impairment in response to a hot environment suggesting that the TIP39-PTH2R system plays a specific role in temperature conservation in a cold environment (Dimitrov et al., 2011). Since temperature sensation was normal in PTH2R KO mice, the PTH2R may play a role in the heat production signal or heat production ability. Both seem to be the case because acute intracerebral PTH2R antagonist administration also impaired the heat production response to a cold environment, though to a smaller extent. In addition, the weight of brown adipose tissue (BAT), and its capacity to increase body temperature were reduced. PTH2Rs in the MnPO seem to be involved in the thermoregulatory action of TIP39 because TIP39 injected locally into the MnPO produced a larger body temperature increase (2°C) for longer periods of time than injection of the same amount of TIP39 into the lateral ventricle. Furthermore, local injection of TIP39 into the dorsomedial hypothalamic nucleus had no effect on the body temperature. The MnPO as a site of action is consistent with its high density of TIP39 terminals and PTH2R immunoreactivity (Faber et al., 2007; Dimitrov et al., 2011) as well as with the known role of MnPO neurons in the control body temperature (Baffi and Palkovits, 2000; Bratincsak and Palkovits, 2004) *via* descending systems regulating BAT thermogenesis and cutaneous vascular tone (Morrison and Nakamura, 2011). The action of TIP39 on the HPA axis and in central thermoregulation allows associations with fever. Although no data are available in this regard, this potential role of the TIP39-PTH2R system will be an interesting line of research in the future.

### Nociceptive functions

The TIP39-PTH2R system may play a role at several levels of nociceptive processing. Its role in nociceptive sensation and spinal cord processing has been revealed by intrathecal administration of TIP39 and antagonizing its actions with the injection of an anti-TIP39 antibody (Dobolyi et al., 2002). Intrathecal injection of TIP39 stimulated a dose-dependent nocifensive response, caudally directed scratching, biting, and licking and decreased the tail-flick and paw-pressure withdrawal latencies. Intrathecal injection of the TIP39 antibody increased the response latency in the thermal tail-flick assay and in the paw-pressure test, corresponding to decreased sensitivity (Dobolyi et al., 2002). These actions are likely related to the facilitation of nociceptive transmission from the DRG to neurons in the spinal cord dorsal horn as intense PTH2R immunoreactivity is present in superficial layers of the spinal cord dorsal horn where most nociceptive afferents terminate (Wang et al., 2000).

The TIP39-PTH2R system may also be involved in the supraspinal regulation of pain processing. Intracerebroventricular injection of TIP39 reduced the latencies in tail-flick and hotplate tests while injection of a PTH2R antagonist had the opposite, anti-nociceptive effect in these tests as well as in the formalin test (Dimitrov et al., 2010). Furthermore, TIP39 and PTH2R KO mice also demonstrated reduced nociceptive responses in these tests, arguing for a pro-nociceptive function of endogenous TIP39 *via* the PTH2R (Dimitrov et al., 2010). These findings are consistent with the distribution of TIP39 and the PTH2R in a variety of brain regions known to be involved in the processing of nociceptive information, including the nucleus of the solitary tract, the parabrachial nuclei, the periventricular gray, the midline thalamic nuclei, the PVN, and the insular and infralimbic cortices. These areas are thought to be components of autonomic-limbic pain-related pathways including the ascending reticular activating system (Benarroch, 2006).

### Regulation of postpartum events in mothers

TIP39 expression is decreased in neurons in both the subparafascicular area and the MPL during the period of pubertal development in rat (Dobolyi et al., 2006b). Since the level of the PTH2R did not decrease, it was hypothesized that TIP39 is induced under some circumstances to act on the already available PTH2Rs. Indeed, a dramatic increase in the TIP39 mRNA levels was demonstrated in the postpartum period using real-time PCR. Upon removal of the pups, the level of TIP39 mRNA decreased to its basal level. *In situ* hybridization histochemistry confirmed the induction of TIP39 and revealed that within the subparafascicular area, TIP39 neurons in the PIL but not in the PVG demonstrated an increase in TIP39 expression (Cservenak et al., 2010). The elevated mRNA of TIP39 in the PIL and MPL is translated into increased peptide levels, as demonstrated by immunohistochemistry (Varga et al., 2008; Cservenak et al., 2010). The functional significance of the elevated TIP39 was tested on the release of prolactin because pathway transections revealed projection of TIP39 neurons in the PIL toward tyrosine hydroxylase-containing neurons in the mediobasal hypothalamic regions known to regulate prolactin secretion. Retrograde labeling in nulliparous female rats also demonstrated a projection of subparafascicular TIP39 neurons to the arcuate nucleus (Szabo et al., 2010). In rodents, removal of the pups from the dams for 4h results in a decrease in prolactin level, which is in turn dramatically increased upon the return of the litter and the immediate onset of nursing. Injection of a PTH2R antagonist into the lateral ventricle 5 min before uniting the mothers with pups potently and dose-dependently inhibited suckling induced prolactin release in the rat (Cservenak et al., 2010). The physiological significance of this is supported by the observation that in a similar pup removal/return paradigm the weight increase (a measure of milk consumed) of pups suckling PTH2R KO mice was reduced 30 min after the onset of nursing as compared to pups suckling WT mice (Coutellier et al., 2011b). Also consistent with less effective suckling by PTH2R KO dams, pups reared by PTH2R KO mice had a lower body weight at the time of weaning than pups reared by WT mice (Coutellier et al., 2011b). To eliminate the effect of the genotype of the pups from the analysis, WT females were mated with PTH2R KO males while PTH2R KO females were mated with WT males so that all pups were heterozygous in these experiments (Coutellier et al., 2011b).

Additional influences of subparafascicular TIP39 neurons on reproductive neuroendocrine function cannot be excluded. TIP39 fibers and PTH2Rs are ideally positioned to affect gonadotropin-releasing hormone (GnRH) neurons, whose activity is suppressed during lactation. In addition, the TIP39-PTH2R neuromodulator system might also play a role in conveying the effect of suckling on other systems adapted in the postpartum period. PTH2Rs in the preoptic area, the lateral septum, and the periaqueductal gray could be involved in the control of maternal behaviors (Dobolyi, 2011). Emotional changes that take place in the postpartum period could also be affected by TIP39 based on the localization of the TIP39-PTH2R system in the infralimbic cortex, the medial, and central amygdaloid nuclei, the amygdalo-hippocampal transitional zone, the premamillary nuclei, the ventral subiculum, and the periaqueductal gray, which are parts of the circuitry of reproductive and emotional regulation (Lonstein and Stern, 1997; Lin et al., 1998; Li et al., 1999; Simerly, 2002; Numan and Insel, 2003; Hasen and Gammie, 2005).

## Possible Models for Actions *via* the TIP39-PTH2R System

### The activation of TIP39 neurons

There is limited information available showing that TIP39 neurons are activated in stress situations. At present, only the effects of an h-long high intensity noise stress have been reported. Medial paralemniscal and posterior intralaminar TIP39 neurons demonstrate Fos induction together with CRH neurons in response to noise stress, suggesting that TIP39 neurons could be involved in the transmission of acoustic stress derived information to CRH neurons in the PVN (Palkovits et al., 2009). In agreement with these findings, medial paralemniscal and possibly also posterior intralaminar TIP39 neurons have afferent neuronal connections with the primary auditory cortex and the external cortex of the inferior colliculus (Varga et al., 2008) providing the anatomical basis for an auditory influence on the CRH neurons in the PVN.

Cold exposure but not warm ambient temperature induced *c-fos* in some PVG neurons (Kiyohara et al., 1995; Miyata et al., 1995; Baffi and Palkovits, 2000; Bratincsak and Palkovits, 2004) providing the possibility that TIP39 neurons in this region could be activated by cold exposure leading to both stress responses and temperature regulation. It has also been reported that the *c-fos* expression in the PVG significantly outlasts the cold exposure (Miyata et al., 1995), suggesting that it may have a role in the maintenance of homeostasis during adaptation to cold stress (Baffi and Palkovits, 2000).

The PVG is a site of stimulation-induced analgesia (Rhodes and Liebeskind, 1978; Peschanski and Mantyh, 1983). Potent analgesia is obtained in rats following electrical stimulation in the gray matter surrounding the caudal portion of the third ventricle and the midline area of the caudal thalamus that is comparable to that produced by stimulation of the caudal periaqueductal gray. Analgesia outlasts the period of brain stimulation, and is not due to a generalized motor debilitation of the animal (Rhodes and Liebeskind, 1978). In addition, some neurons in the area are activated by noxious stimuli (Dong et al., 1978; Sugiyama et al., 1992) providing the possibility that TIP39 neurons in this region could be activated by noxious stimuli leading to both stress and nociceptive responses.

The TIP39-PTH2R neuromodulator system is present in several brain areas that are activated in males following mating (Sachs and Meisel, 1988; Coolen et al., 1997; Veening and Coolen, 1998) including the PIL, the medial preoptic nucleus, the posteromedial part of the medial subdivision of the bed nucleus of the stria terminalis, and the posterodorsal subdivision of the medial amygdaloid nuclei. In addition, the PIL, which contains TIP39 neurons, has also been implicated in sexual functions *via* the facilitation of copulatory behavior by current injection into the area (Shimura and Shimokochi, 1991), by the decreased sexual behavior following lesion in the area (Maillard and Edwards, 1991), and by the demonstration of the activation of neurons in the area following sexual behavior using the Fos technique in rats (Coolen et al., 1997) and imaging techniques in human (Holstege et al., 2003). Indeed, TIP39 neurons in the PIL have been shown to exhibit *Fos* expression following ejaculation (Wang et al., 2006a). This suggests that these TIP39 neurons are part of the afferent circuits that process genital-somatosensory information related to ejaculation contributing to mating and mating-induced changes in reproductive behaviors.

Another reproductive influence reaching TIP39 neurons in the PIL was reported in mother rats. Dams deprived of their pups for a day demonstrated *c-fos* expression in the PIL but not in the PVG in response to reunion with their litter (Cservenak et al., 2010). Almost all TIP39 neurons in the PIL were involved in this response. Since the pups start to suckle very soon after they are returned to their mothers, it was hypothesized that suckling results in the activation of TIP39 neurons in the PIL (Cservenak et al., 2010).

### The potential mechanisms of action of TIP39

**Figure 3:**
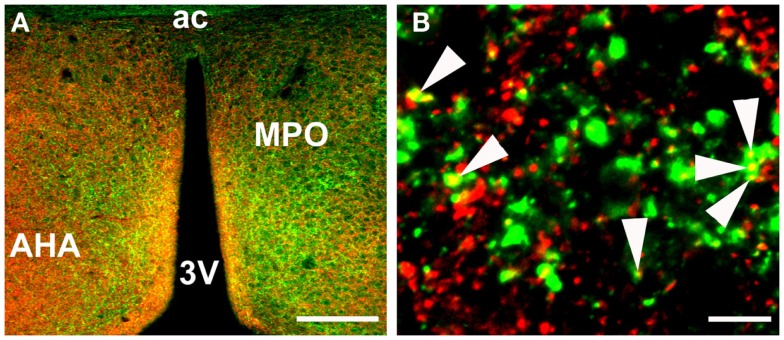
**Colocalization of PTH2R fibers and VGluT2 punctae in the anterior hypothalamus as detected by immunohistochemistry**. **(A)** a low magnification image of PTH2R (green) and VGluT2 (red) expression in the region of the medial preoptic area. **(B)** a high magnification image of PTH2R/VGluT2 colocalization, where some of the colocalized points are indicated with arrowheads. Abbreviations: 3V, third ventricle; ac, anterior commissural; AHA, anterior hypothalamic area; MPO, medial preoptic nucleus of the hypothalamus. Scale bars = 200 μm for A and 10 μm for B.

**Figure 4:**
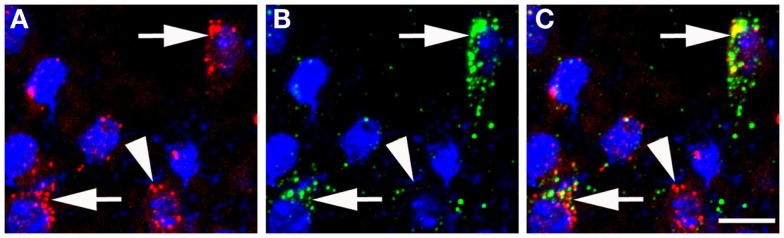
**Colocalization of VGluT2 mRNA and PTH2R mRNA in the anterior hypothalamus as detected by fluorescent *in situ* hybridization**. **(A)** VGluT2 mRNA (red puncta) over cell nuclei (blue, DAPI). **(B)** PTH2R mRNA (green puncta) over cell nuclei (blue, DAPI). **(C)** a merged image, where the arrows point to cells expressing both PTH2R/VGluT2 mRNAs and the arrowhead points to a neuron with only VGluT2 signal. Scale bar = 10 μm.

There are no indications at present that TIP39 would have any action apart from activating the PTH2R. The PTH2R elevates cAMP and in some cells also increases Ca^2+^ levels (Goold et al., 2001; Della Penna et al., 2003) suggesting an excitatory influence of TIP39 on the target cells. In some cases, the PTH2R may be located in cell bodies and dendrites, there is evidence for this for the somatostatin neurons in the rat periventricular nucleus. Most typically, the PTH2R may be located in axon terminals. Apart from the somatostatin terminals in the median eminence (Dobolyi et al., 2006a), a presynaptic location of the PTH2R at excitatory synapses has been demonstrated in several hypothalamic areas (Figure 3). In the PVN, vesicular glutamate transporter 2 (VGluT2)-containing terminals closely apposed to CRH neurons were shown to also contain the PTH2R (Dimitrov and Usdin, 2010). In addition, the close apposition by TIP39-containing terminals suggested that TIP39 from the subparafascicular area might also contribute to the activation of CRH neurons by increasing the efficacy of the excitatory input to the CRH neurons (Dimitrov and Usdin, 2010). In a similar fashion, median preoptic neurons projecting to the dorsomedial hypothalamic nucleus for the potential transfer of thermoregulatory information were also suggested to be modulated by TIP39 *via* presynaptic excitation of their afferents (Dimitrov et al., 2011). Expression of the PTH2R by glutamatergic neurons in several brain regions is supported by data from *in situ* hybridization histochemistry (Figure 4). In other cases, the effect of TIP39 on inhibitory neurons has also been postulated based on functional data (Sugimura et al., 2003; Cservenak et al., 2010). Thus, the inhibition of AVP neurons and dopaminergic neurons is expected from the actions of TIP39 on the serum levels of AVP and prolactin, respectively. This inhibition could be carried out *via* potentiation of excitatory inputs to inhibitory interneurons acting on the AVP and dopaminergic neurons, respectively.

## Conclusion

**Figure 5:**
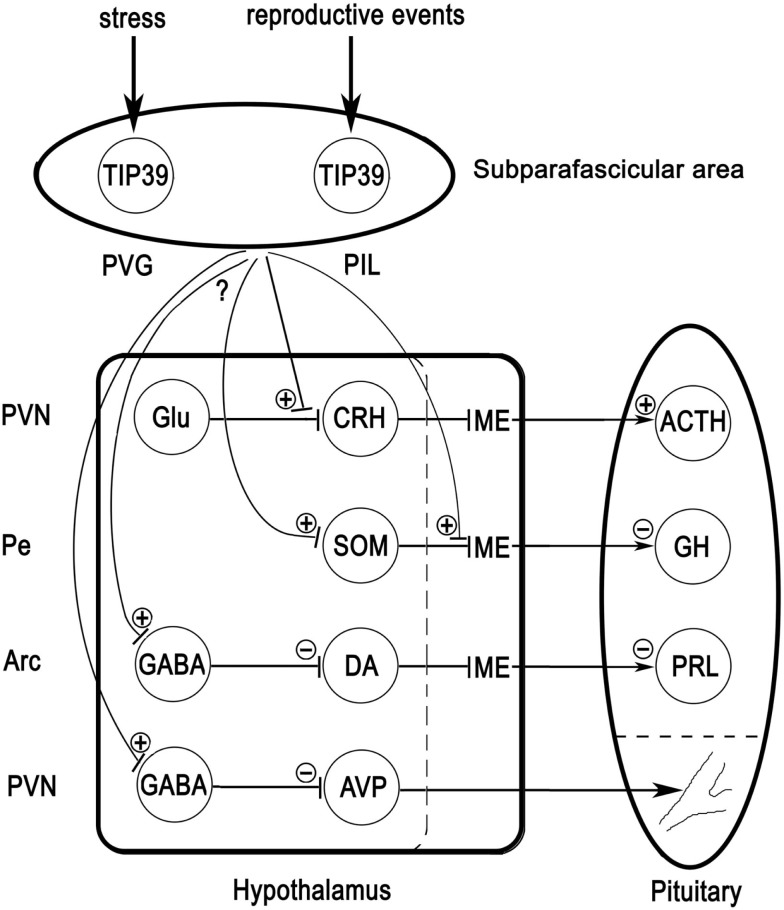
**Potential mechanisms of actions of TIP39 *via* the PTH2R**. TIP39 neurons in the PVG receive information on threats to homeostasis while those in the PIL receive various inputs related to reproduction. Axon terminals arising from these TIP39 neurons may facilitate excitatory synapses by presynaptic actions. A well-documented example is the action of TIP39 on glutamatergic synapses terminating on CRH neurons in the PVN. Another possible route to influence hypothalamic hormone release is *via* terminals of hypophysiotropic neurons in the median eminence (ME) as has been suggested for somatostatin. Neurons that express PTH2Rs in their cell bodies may be directly affected by TIP39. Some actions of TIP39 including those on prolactin and AVP release, seem to be mediated by inhibitory neurons. TIP39 terminals might innervate cell bodies of GABAergic cells or alternatively, presynaptic terminals leading to the activation of inhibitory neurons (not shown). Additional abbreviations: ACTH, adrenocorticotropin; Arc, arcuate nucleus; DA, dopamine; GH, growth hormone; Glu, glutamate; Pe, periventricular hypothalamic nucleus; PRL, prolactin; SOM, somatostatin.

The TIP39-PTH2R neuromodulator system may play an important role in the regulation of several different aspects of neuroendocrine functions. TIP39 neurons in the PVG may be stimulated by stress inputs compromising the homeostasis of the animal while TIP39 neurons in the PIL may be stimulated by various reproductive events (Figure 5). Additional, as yet unexplored inputs to the TIP39 neurons are also plausible. These TIP39 neurons in the posterior thalamus relay their input toward neurons located in neuroendocrine and limbic brain areas to exert direct and indirect effects on neuroendocrine systems. So far, substantial evidence is available for the involvement of the TIP39-PTH2R system in the regulation of corticosterone, AVP, and prolactin. These actions of TIP39 suggest that the PTH2R may be a future target to develop drugs for the treatment of some neuroendocrine abnormalities and disorders.

## Conflict of Interest Statement

The authors declare that the research was conducted in the absence of any commercial or financial relationships that could be construed as a potential conflict of interest.
